# Decoding AI for transplantation: many models, one patient: why there is no single “AI for transplantation” – and what to ask instead

**DOI:** 10.3389/ti.2026.17554

**Published:** 2026-09-30

**Authors:** Olivier Aubert, Esteban Cortes Garcia, Silvia Pineda, Thomas Neyens, Nina Pilat

**Affiliations:** 1 Executive Editor, Transplant International; 2 Department of Kidney and Metabolic Diseases, Transplantation and Clinical Immunology, Necker Hospital, Assistance Publique - Hôpitaux de Paris, Paris, France; 3 Université Paris Cité, Institut National de la Santé et de la Recherche Médicale U1151, Centre National de la Recherche Scientifique Unité Mixte de Recherche 8253, Institut Necker Enfants Malades, Paris, France; 4 Statistical Editor, Transplant International; 5 Statistics and Data Science Department, Universidad Complutense de Madrid, Madrid, Spain; 6 Leuven Centre for Biostatistics, KU Leuven, Leuven, Belgium; 7 Data Science Institute, Hasselt University, Hasselt, Belgium; 8 Deputy Editor-in-Chief, Transplant International; 9 Department of Cardiac and Thoracic Aortic Surgery, Medical University of Vienna, Vienna, Austria; 10 Centre for Biomedical Research and Translational Surgery, Medical University of Vienna, Vienna, Austria

**Keywords:** artificial intelligence, multimodal models, rejection, statistics, transplantation

## Opening vignette

Consider a routine follow-up with a kidney transplant recipient: You are sitting with your patient presenting with an elevated serum creatinine. To assess potential allograft rejection or injury you do not reach for a single piece of information. You look at the trend rather than one value, you check the proteinuria, you recall the donor-specific antibodies from last year, you open the most recent biopsy results and you weigh all of it against everything you know about this particular patient. In a few minutes you have combined five different kinds of information into one judgement, almost without noticing. This ordinary act–holding many sources together and deciding what they mean as a whole–turns out to be the single hardest thing for artificial intelligence to do. The current landscape of AI tools in transplantation largely features fragmented, siloed architectures. Most of these tools read a single type of data–the biopsy, the transcriptomic signature, or the cfDNA value alone–and are built to answer one specific diagnostic or prognostic question.

## There is no single “transplant AI”

With the launch of this inaugural piece, we proudly introduce our new educational series, “Decoding AI for Transplantation.” This opening contribution is intended to paint the overarching landscape of the field, establishing a baseline framework before subsequent educational pieces look closely at specific computational methods, novel algorithmic approaches, and pressing ethical concerns.

A pervasive challenge in contemporary medical discourse is the tendency to evaluate “Artificial Intelligence” as a single, monolithic entity, like a uniform “black box” expected to revolutionize clinical care overnight. In reality, there is no singular, all‐encompassing AI tool for transplantation; instead, the technology consists of highly narrow algorithms (or “AI tools”) designed for a specific purpose, each reading a particular kind of data [1, 2]. The fundamental clinical skill this series aims to cultivate, therefore, is not the capacity to critique “AI” in the abstract, but rather the practical ability to differentiate between these disparate technologies. This analytical categorization can largely be achieved by interrogating two fundamental questions: first, *what specific modality of data does the tool read* and second, *what precise clinical objective is it engineered to fulfil?*

## What does it read, and what can it do?

Take the two questions in turn, because together they locate almost any tool you will meet (Figure 1).

**FIGURE 1:**
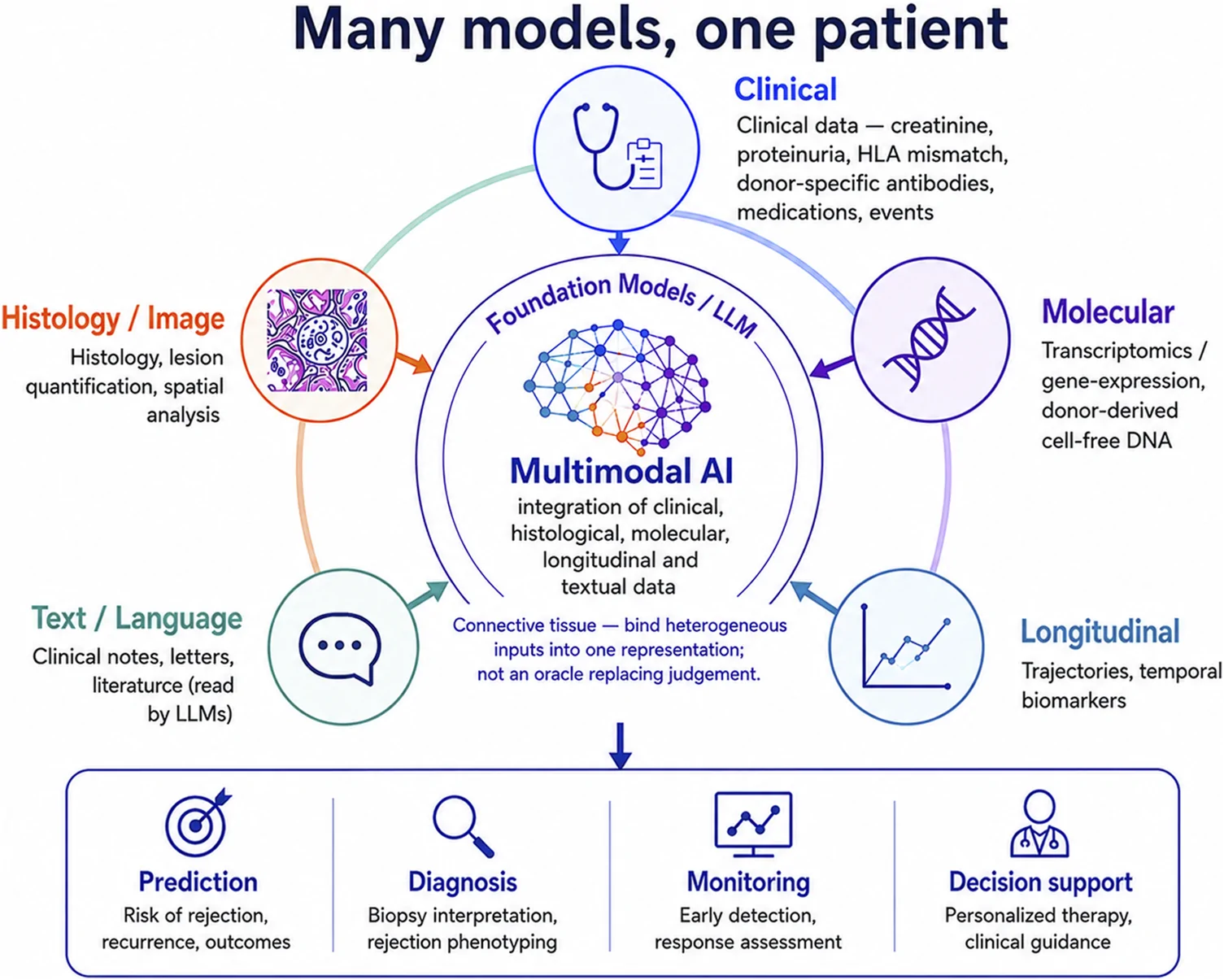
Around the edge are the five data modalities a tool may read–clinical, histological/image, molecular, longitudinal and textual; along the bottom are the clinical tasks it may perform–diagnosis, monitoring, prediction and decision support. Most tools occupy a single modality for a single task; some already combine several sources into a multimodal model (centre), where foundation models and large language models increasingly act as the connective tissue binding heterogeneous inputs into one representation. There is no single “transplant AI”–only many tools at different points on this map, moving unevenly toward the integration a clinician performs at the bedside.

The first question is simple: what kind of data does the tool read? We call this its modality. Some tools read structured clinical data–creatinine, proteinuria, HLA mismatch, donor-specific antibodies. Others read images, typically the digitized biopsy slide [3]. Some read molecular data – the gene-expression signature of a tissue sample [4, 5], or donor-derived cell-free DNA in the blood [6]. Some read longitudinal data, following how a value moves over months rather than its level on a single day. And some now read free text–notes, letters, the literature–using large language models [7]. Each modality is, in effect, a different language, and most tools speak only one.

The second question is the task–the job the tool performs. A tool may be built to diagnose (what is happening in this graft now?), to screen or monitor (is something developing before it becomes clinically obvious?), to predict (what is the long-term risk?), or to guide a treatment decision. The same modality can serve different jobs, and the same job can be approached through different modalities–which is precisely why the word “AI” tells you so little on its own.

Placing real transplant tools on these two axes makes the point concrete. Digital-pathology models read images to help diagnose rejection on biopsy [3]. Transcriptomic classifiers read molecular data for the same diagnostic question [4, 5], by an entirely different route. Donor-derived cell-free DNA, increasingly embedded in multimodal screening tools, reads the blood to monitor for subclinical rejection before a biopsy is taken. A risk score such as the iBox reads clinical, histological and immunological data together to predict long-term graft survival [8]–the first of our four tools to combine modalities rather than rely on one. Four tools, four positions on the map, and only by naming each tool’s data and task can you judge what it offers, and what it cannot.

## The illusion of completeness: unimodal precision vs. clinical synthesis

Categorizing a tool by its data input and clinical function reveals a critical limitation that a single headline metric can easily hide: most algorithms capture only a narrow slice of the total clinical picture. The biopsy-reading model has never seen the cell-free DNA result; a purely clinical risk score knows nothing of the histology. Each model can be excellent within its specific (diagnostic) domain and is, by design, blind to everything outside it.

That is the reverse of what a clinician does. At the bedside you are the integrator: the value of your judgement comes not from any one number but from seeing the big picture and holding the individual results together–the rising creatinine made meaningful by the proteinuria, the antibodies reinterpreted in the light of the biopsy. A tool that returns one confident result from one source is, in this sense, doing something you cannot; it answers a narrower question–often very well, but a narrower one. A narrow answer delivered with confidence is easy to mistake for a complete one.

To be fair, some tools already reach beyond a single modality. But these still remain narrow next to the synthesis a clinician performs without thinking. The honest description of the field is a spectrum: many single-modality tools, a growing few that combine two or three sources, and none that yet sees the whole patient, but then neither does the clinician alone, and multimodal integration is precisely the path toward seeing more than either the eye or a single tool can on its own.

**Box 1 – Decoding AI: five fundamental questions**

1. What kind of data does this tool read–clinical, image, molecular, longitudinal, or text?
2. What is its job–diagnosis, screening or monitoring, prediction, or decision support?
3. Does it read one modality or combine several–and if it integrates, how does it cope when a source is missing?
4. Was it tested in patients and centre other than the ones it was built on?
5. Does it change a decision or improve an outcome–or only describe a risk you already sensed?

**Box 2 - Key takeaways and glossary**

**Three takeaways**

1. There is no single “transplant AI”: the field is many tools, each defined by the data it reads and the clinical job it does.
2. Most tools see only a slice of the patient; some already combine modalities, but none yet matches the integration a clinician performs at the bedside.
3. The direction is integration (multimodal AI) - already underway, genuinely hard, and to be read more critically as it grows, not less.

**Glossary**

**Data modality**. The kind of information a tool reads - clinical numbers, biopsy images, molecular signals, time trajectories, or text.

**Task**. The clinical job a tool performs - diagnosis, screening/monitoring, prediction, or decision support.

**Multimodal AI**. A model that combines several data types into one output, rather than relying on a single source.

**Foundation model/large language model (LLM)**. A large, general-purpose model (often trained on text) that can take in varied inputs and be adapted to many tasks.

**External validation**. Testing a tool in patients or centres other than those it was developed on, to see whether it travels.

## Putting the patient back together–and why this is so difficult

The direction of travel follows from all this, and it is the ambition behind the centre of Figure 1: to combine these tools, feeding clinical, histological, molecular and longitudinal information into models that produce one integrated picture. This is what multimodal AI means–and the principle is not speculative.

It is worth understanding why this frontier is genuinely hard, because this is where many promising tools stumble. Different modalities live on different scales and timeframes: a slide, a single blood value and a years-long trajectory are not naturally comparable and forcing them into one model confronts us with a real problem, not a formality. Sources are often missing–no biopsy this visit, the anti-HLA DSA result not yet back–and a model trained on complete data can behave unpredictably without them [9]. Unlike traditional statistics, which offers a wide range of flexible methods for missing data [10], AI methodology is not yet at that stage; the more sources a model blends, the harder it becomes to see why it reached its conclusion, exactly when you most want to know. This is the role increasingly proposed for foundation models and large language models: not as oracles that replace judgement, but as connective tissue able to take in very different kinds of input and help bind them–a useful job, and a difficult one.

The holy grail, stated simply, would be to train computational models to replicate what clinicians already perform instinctively: to evaluate the patient holistically rather than viewing an isolated organ system or a singular assay. In reality we should not try to develop machine learning methods that replicate what clinicians do, but provide better tools to help clinicians make better decisions. This conceptual thread unifies this article series and underlines the message visualized in Figure 1, illustrating a trajectory that moves from the fragmented, highly specialized tools of today toward a fully integrated clinical paradigm tomorrow.

## A framework for critical appraisal

For the reader, this reframing turns an intimidating field into a set of answerable questions. Faced with any “AI in transplantation” paper or product, the first move is not to ask whether “AI works”, but to locate the tool. The first two questions have been introduced: what data does it read, and what job does it do? Three further questions complete the appraisal: how much does it integrate, how was it tested, and does it challenge my decision [7, 11–13]? A tool that reads one modality for one task can be excellent and still be only a part of the picture. A tool that claims to integrate many should be held to a higher standard, not a lower one, because its reach is wider and its failures quieter. And the ordinary questions never disappear–was it validated elsewhere, does it change a decision, does it help the patient. There is no single “transplant AI” to adopt or dismiss; there are many tools, each to be read on its own terms. Embracing this complexity is a more demanding approach, but ultimately a more useful one. In the end the question is not whether AI can replace our judgement but how we can use AI to make the best possible judgement to help patients.
